# Everolimus as a therapeutic option in refractory epilepsy in children with tuberous sclerosis: a systematic review

**DOI:** 10.1055/s-0042-1758442

**Published:** 2023-03-02

**Authors:** Catarina Ester Gomes Menezes, Débora Lopes dos Santos, Erick Santos Nery, Evelin Duarte Serpa, Lécio Aragão Souza Morais, Lucas Santana Dutra, Marcos Baruch Portela Filho, Julieta Sobreira Goes

**Affiliations:** 1 Universidade do Estado da Bahia, Departamento de Ciências da Vida, Salvador BA, Brazil.; 2 Escola Bahiana de Medicina e Saúde Pública, Departamento de Medicina, Salvador BA, Brazil.

**Keywords:** Tuberous Sclerosis, Epilepsy, Everolimus, TOR Serine-Threonine Kinases, Child, Esclerose Tuberosa, Epilepsia, Everolimo, Serina-Treonina Quinases TOR, Criança

## Abstract

**Background** Tuberous sclerosis (TS) is a multisystem genetic disease in which epilepsy is a frequent manifestation and is often difficult to control. Everolimus is a drug with proven efficacy in the treatment of other conditions related to TS, and some evidence suggests that its use benefits the treatment of refractory epilepsy in these patients.

**Objective** To evaluate the efficacy of everolimus in controlling refractory epilepsy in children with TS.

**Methods** A literature review was conducted in the Pubmed, BVS, and Medline databases, using the descriptors *Tuberous sclerosis* , *Children* , *Epilepsy* , and *Everolimus* . Original clinical trials and prospective studies published in Portuguese or English in the last decade that evaluated the use of everolimus as an adjuvant therapy in the control of refractory epilepsy in pediatric patients with TS were included.

**Results** Our search screened 246 articles from electronic databases, 6 of which were chosen for review. Despite the methodological variations between the studies, most patients benefited from the use of everolimus to control refractory epilepsy, with response rates ranging from 28.6 to 100%. Adverse effects were present in all studies leading to dropouts of some patients; however, the majority were of low severity.

**Conclusion** The selected studies suggest a beneficial effect of everolimus in the treatment of refractory epilepsy in children with TS, despite the adverse effects observed. Further studies involving a larger sample in double-blind controlled clinical trials should be performed to provide more information and statistical credibility.

## Resumo

**Antecedentes** A esclerose tuberosa (ET) é uma doença genética multissistêmica na qual a epilepsia é a manifestação neurológica mais frequente, sendo muitas vezes de difícil controle. O everolimo é uma droga com eficácia comprovada no tratamento de outras condições relacionadas à ET, e indícios sugerem benefícios de seu uso também no controle da epilepsia refratária nesses pacientes.

**Objetivo** Avaliar a eficácia do everolimo no controle da epilepsia refratária em crianças com ET.

**Métodos** Revisão de literatura nas bases de dados Pubmed, BVS e Medline, utilizando os descritores *Tuberous sclerosis* , *Children* , *Epilepsy* e *Everolimus* . Incluíram-se ensaios clínicos originais e estudos prospectivos publicados em português ou inglês na última década e que avaliassem o uso do everolimo como terapia adjuvante no controle da epilepsia refratária em pacientes pediátricos com ET.

**Resultados** Nossa busca rastreou 246 artigos nas bases de dados, dos quais 6 foram escolhidos para a revisão. Apesar das variações metodológicas entre os estudos, a maioria dos pacientes tiveram benefício no uso do everolimo para controle da epilepsia refratária, com taxas de resposta variando entre 28.6 e 100%. Os efeitos adversos estiveram presentes em todos os estudos, levando à desistência de alguns pacientes, contudo a maioria foi de baixa gravidade.

**Conclusão** Os estudos selecionados sugerem efeito benéfico do everolimo no tratamento da epilepsia refratária em crianças com ET, apesar dos efeitos adversos observados. Novos estudos envolvendo uma amostra maior em ensaios clínicos controlados duplo-cegos devem ser realizados para fornecer mais informações e credibilidade estatística.

## INTRODUCTION

The tuberous sclerosis complex (TSC) is a multisystem syndrome caused by mutations in the TSC1 or TSC2 gene, which are responsible, respectively, for decoding the hamartin and tuberin proteins. As they are tumor suppressor genes, mutation of TSC1 or TSC2 leads to loss of inhibitory influence on the cell cycle by hyperactivation of the mTOR protein (mammalian target of rapamycin), culminating in disordered proliferation of tissues and formation of hamartomas—benign tumor-like lesions—in several tissues, in addition to other manifestations such as neurodevelopmental disorders and epilepsy. 1 2 3 4

Therefore, mTOR has become a potential target for pharmacological therapy of TSC. The first mTOR inhibitor drug approved for treating TSC in the United States and Europe was everolimus (RAD001), and its use for the treatment of some manifestations related to TSC, such as subependymal giant cell astrocytoma and renal angiomyolipoma, is already well established in the international literature. Some studies also demonstrate positive results in the use of everolimus for the treatment of refractory epilepsy associated with TSC; however, there is still no formal recommendation or consensus in literature. 3 5

Epilepsy is the most common neurological manifestation in TSC, and it is often difficult to control as most currently available therapeutic options are ineffective and/or associated with serious side effects. Approximately 80 to 85% of patients with TSC have at least one epileptic seizure in their lifetime—most within the 1st year of life—and many develop epilepsy. 6 7 8

In 2010, Kwan et al. described refractory epilepsy as the persistence of epileptic seizures after the use of 2 anticonvulsant medications, specifically chosen for the type of epilepsy presented, used as monotherapy or in association. 9 The presence of refractory epilepsy is related to an increased risk of cognitive impairment, especially in children under 3 years of age. Therefore, the search for therapies capable of reducing the frequency of epileptic seizures and controlling refractory epilepsy is essential to minimize and potentially prevent the negative impacts of uncontrolled epilepsy in the child population, in addition to reducing mortality. 9 10

In the context of TSC-related epilepsy, the use of mTOR inhibitor drugs, such as everolimus, represents a possibility for a treatment for refractory epilepsy, targeting the genetic cause. 5 11 Thus, this systematic review aims to evaluate the efficacy of everolimus in controlling refractory epilepsy in pediatric patients with tuberous sclerosis.

## METHODS

### Study design

This article is a systematic review that addresses the therapeutic use of everolimus in the treatment of pediatric patients with refractory epilepsy associated with tuberous sclerosis.

### Search strategies

A systematic review of the literature was conducted through the selection of articles in the electronic databases Pubmed, Virtual Health Library, and Medline, on February 4, 2021. For the search, descriptors were taken from Medical Subject Heading (MeSH) and Descriptors in Health Science (DeCS). The terms used were *Tuberous sclerosis* , *Children* , *Epilepsy,* and *Everolimus* .

A period of 10 years prior to the search date was defined as the limit for selecting articles. The present review was submitted on the International Prospective Register of Systematic Reviews (PROSPERO) platform, and the search strategies and selection of studies followed the preferred reporting items for systematic reviews and meta-analyses (PRISMA) protocol.

### Inclusion and exclusion criteria

Original studies such as clinical trials or prospective cohorts published in Portuguese and English, between January 2011 and January 2021, which evaluated the use of everolimus as adjunctive therapy in the control of refractory epilepsy in pediatric patients (between 0 and 21 years) with TSC were included. Cross-sectional studies, review articles, case reports, comments, correspondence, and articles published in other languages were excluded.

## RESULTS

Two hundred forty-six articles were found in the chosen electronic databases. Of these, 233 were excluded after reading the title and abstract, as follows: 122 for repetition in the databases, 4 for not being available in Portuguese or English, 61 for not presenting a study design compatible with the inclusion criteria, and 46 for not addressing the proposed topic.

Only 13 articles were eligible for full-text reading. At this stage, we excluded 6 studies for not being compatible with the inclusion criteria, such as including participants who were not from the children's group or using databases of larger-scale studies previously included. In the end, six articles met all the inclusion criteria and were selected for this systematic review.

Of the 6 articles selected at the end, 5 had small samples (< 20 patients), 4 were prospective, nonrandomized, open-label studies, 12 13 14 15 and 2 were double-blind, randomized studies. 16 17 In Table 1 , we summarize the main features of the 6 studies selected.

**Table 1:** Characteristics of six articles included in this review

Authors and year of publication	Type of study	Sample	Sex	Main adverse effects	Response rate	Dosage
Kotulska et al., 17 2013	Prospective, double-blind, placebo-controlled, multicenter phase 3 study	5	25% female; 75% male	Stomatitis, diarrhea, and elevation of alanine transaminase	60%	4.5 mg/m ^2^ (2.53–8 mg/m ^2^ )
Wiegand et al., 12 2013	Open-label case series	6	57.1% female; 42.9% male	Upper respiratory, gastrointestinal tract infections, and exanthema	33.3%	1 mg/m ^2^
Krueger et al., 13 2016	Prospective, non-randomized, open-label clinical trial	Initial phase: 20 Extension phase: 14	50% female; 50% male	Infections and stomatitis	93%*	0.34 mg/kg/d (0.06–1.02)
Curatolo et al., 16 2018	Multicenter, double-blind, randomized, phase 3 study	Core phase: 299 Extension phase: 294	Core Phase: 47% female and 53% male Extension Phase: 46.5% female and 53.5% male	Pyrexia, diarrhea, stomatitis, mouth ulceration, upper respiratory tract infection, nasopharyngitis, cough, and vomiting	Younger subgroup: 48.9%. Older subgroup: 47.2% **	< 10 years old: 6–9 mg/m ^2^ 10–18 years old: 5–8 mg/m ^2^
Samueli et al., 14 2018	Prospective open-label observational study	4	100% male	Hypertriglyceridemia, recurrent viral infections, impetigo, and pharyngitis	***	4.5 mg/m ^2^
Svarrer et al., 15 2019	Prospective open-label observational study	4	50% female; 50% male	Exanthema, stomatitis, diarrhea, and neutropenia	100%	5–9 mg/m ^2^

Notes: *Response rate at the end of extension phase (year 4); **Response rate at the end of extension phase (weeks 42–54); ***The article did not provide the response rate, and it was not possible to calculate it based on the available data. However, at the end of the study, two patients had remission of seizures, one had focal seizures, and one maintained infantile spasm. Three patients switched from hypsarrhythmia patterns to focal epileptic discharges, and one patient maintained hypsarrhythmia.

Kotulska et al. 17 evaluated the long-term effects of everolimus on epilepsy in eight children under three years of age with subependymal giant cell astrocytoma associated with TSC. Three of these patients were seizure-free at baseline with the use of one or two antiepileptic drugs each and were evaluated considering only the tumor size after the use of everolimus, being, therefore, excluded from this review. The seizures were recorded daily by the caregivers in a seizure diary. After a mean follow-up of 35 months, 1 child with drug-resistant epilepsy had complete remission of seizures with the use of everolimus, and 2 others had ≥ 50% reduction in seizures, which represents a response rate of 60% considering only the 5 patients who were not seizure-free at baseline. The initial everolimus dose was 4.5 mg/m ^2^ , later adjusted to reach a serum concentration of 5 to 15 ng/mL, resulting in final doses between 2.53 and 8 mg/m ^2^ . All patients developed at least one adverse effect (AE), and the most common were upper airway infections (8), stomatitis (7), exanthema (4), hyperlipidemia (3), leukopenia (2), and decreased serum fibrinogen levels (2), with the last two appearing more severely in one patient each. In addition, five patients manifested stomatitis classified as a grade III AE according to the common terminology criteria for adverse events (CTCAE).

The study carried out by Wiegand et al. 12 in 2013 was an open-label case series with 7 infants with intractable epilepsy who required a mean of 6.9 antiepileptic drugs before the beginning of the study. Patients were treated with everolimus for 36 weeks at an initial dose of 1 mg/m ^2^ , which was gradually increased until reaching a serum concentration of 5 to 10 ng/ml. One patient withdrew from the study after 35 days of everolimus use due to a relapsing facial rash (common terminology criteria for adverse events [CTCAE] grade II). Seizures were documented by the parents using an internet-based seizure diary (EPI-Vista). After a 36-week follow-up, 2 (33.3%) patients had a ≥ 50% reduction in seizures, 2 patients were near-responders, and the other 2 patients had no significant reduction in epileptic seizures. All patients had side effects, the most reported being: upper airway infections (5), increased transaminases (5), mild thrombocytosis (4), gastrointestinal tract infections (4), hyperlipidemia (4), decreased serum chloride (4), increased erythrocytes (3), pyuria (3), nasopharyngitis (2), conjunctivitis (2), otitis (2), pneumonia (1), and stomatitis (1). Two patients manifested upper airway infections (grade III), leading to hospitalization. Patients who continued long-term treatment with everolimus reported a reduction in adverse effects over time, causing no further discontinuation of treatment.

The study published in 2016 by Krueger et al., 13 which was an extension of a previous study from 2013, is divided into a main study and an extension phase that lasted 48 months and is aimed at assess the long-term benefits of everolimus administration in patients with epilepsy secondary to TSC. Electroencephalogram was performed at baseline and at the end of the main treatment phase but did not continue into the extension phase. Caregivers used a standardized diary to record seizure description, duration, and frequency. The response rate was determined based on the clinical ictal semiology recorded in seizure diaries. The study started with 20 patients, and by the end of the main study, 12 (60%) patients had a ≥ 50% reduction in seizures, and 4 (20%) patients were near-responders (25–49% reduction compared to baseline). The extension phase started with 18 patients, but only 14 remained until the end of 48 weeks, with high response rates throughout the extension phase. Response rates were 76%, 75%, 80%, and 93%, respectively, during 12, 24, 36, and 48 months of treatment, and partial responders over the same period were 6%, 13%, 13%, and 0%. Three patients were removed from the study in the extension phase due to loss of efficacy, and one withdrew consent.

When quantifying adverse events related to the use of everolimus, Krueger et al. 13 reported 416 episodes in the main and extension phases, and 72.5% of these were considered related to everolimus use. Among the most common were infections (217), oral/gastrointestinal disorders (114), and constitutional symptoms (27). A total of 94% of patients reported mild or moderate and well-tolerated AEs, and only 12 episodes of serious AEs were observed (2% of all AEs), all of which led to hospitalizations and that resolved without sequelae, such as infections (11) and status epilepticus (1).

Curatolo et al. 16 performed a posthoc analysis of EXIST-3, 18 a multicenter, double-blind, randomized, phase 3 study that provided the highest degree of evidence to date for the use of everolimus as an adjunctive treatment for epilepsy in TSC. The frequency of seizures was recorded by the patients' caregivers in a seizure diary. Patients were randomly assigned (1:1:1) to receive placebo, low-exposure everolimus (3–7 ng/mL) or high-exposure everolimus (9–15 ng/mL). Randomization was performed by age subgroups (< 6 years, n = 104; ≥ 6 years to < 18 years, n = 195), and there was a statistically significant difference between the placebo groups and the medication-exposed groups concerning the response rate and percentage reduction in mean seizure frequency. Most AEs were classified as mild-to-moderate (grades I and II) and resolved with dose reduction or momentary drug interruption.

The study by Curatolo et al. 16 was the one with a pronounced variety of adverse effects. In its initial sample of 299 individuals, the most recurrent signs and symptoms were fever (118), stomatitis (111), diarrhea (89), nasopharyngitis (75), upper airway infections (73), coughing (67), vomiting (63), pneumonia (38), and bronchitis (35). Adverse effects grades III and IV still appear in this study, mainly pneumonia (24) and stomatitis (7). Three deaths were reported: one from pneumonia, one from septicemia, and one from sudden unexpected death in epilepsy but with no direct association to everolimus treatment.

In 2018, the prospective open-label observational study by Samueli et al. 14 gathered 4 male patients aged 6.0 to 12.0 months with West syndrome associated with TSC. For the analysis, the median observation period was 13 months (8–42). Caregivers recorded seizures through seizure diaries. Half of the infants had electroclinical seizure remission after 2 weeks of treatment. At the time of the last observation, half of the participants were still not seizure-free, but in one of these, hypsarrhythmia was resolved, and the EEG showed only focal epileptic discharges. In the other nonresponder, hypsarrhythmia was still present at the end. In addition, three infants showed improvements in neuropsychomotor development. The treatment was well tolerated in all patients, and there was no need for withdrawal. All infants had grade I AEs: mild hypertriglyceridemia (3) and frequent viral infections (2). Two children had contagious impetigo and recurrent pharyngitis, both classified as grade IV AEs, which required treatment interruption for 2 months.

Svarrer et al. 15 led a prospective open-label observational study in 2019 in which all 4 patients had a > 50% reduction in seizure frequency after 12 months of treatment with everolimus. The families provided a weekly seizure diary. The median age was 3.4 years (2.2–7.4), and the starting dose was 5 to 9 mg/m ^2^ . The AEs described were exanthema, stomatitis, diarrhea, and neutropenia, all mild-to-moderate, self-limited and without quantification of affected patients or episodes.

## DISCUSSION

Epilepsy is the most common neurological symptom in patients with TSC, occurring in about 75 to 90% of affected individuals, mainly in the form of infantile spasms (IS). It is also considered to be one of the main causes of mortality in TSC and is associated with several behavioral disorders that reduce the quality of life. 19 20 21 22 Therefore, it is essential to control the occurrence of epileptic seizures as a way to improve quality of life and life expectancy in these patients.

Kotulska et al. 17 was the first to address the efficiency of everolimus in epilepsy control. The authors presented a trial of 5 participants in early childhood, with a median age of 1.67 years (1–2.83) and observed a relevant response rate of 60%. Prior to the treatment with everolimus, adrenocorticotropic hormone (ACTH) was given to three of the five children for seizure control. Adrenocorticotropic hormone was able to control the IS in these children, but the patients continued to suffer from focal seizures, which were later well controlled with everolimus. Adverse effects were tolerable and similar to those observed in older patients. Therefore, this study supports the possible benefit of the mTor inhibitor for this type of seizure.

The study of Wiegand et al. 12 reported a population with a median age of 5 years (2–12), and positive results were obtained regarding the decrease in the frequency of seizures and the increase in seizure-free days. The open-label design of the study and the fact that the epileptic episodes were documented by the parents in a seizure diary are possible biases.

A clinical trial by Krueger et al. 13 followed patients who were given everolimus for a period of 4 years and attested a long-term effectiveness of everolimus in reducing the frequency of epileptic seizures, as well as a good drug tolerability. Another important finding was that focal-onset seizures had a better response to treatment (83% reduction) compared to generalized-onset seizures (41% reduction).

The effect of everolimus in the control of epilepsy amongst four patients with West syndrome was assessed in the study by Samueli et al. 14 Two participants were free of seizures as a result of treatment with everolimus. Children who started using the mTOR inhibitor after 2 to 3 months from the beginning of epilepsy had good control of symptoms, whereas those who started after 4 to 8 months did not. Thus, an important inference from this study is that patients with a reduction in seizure frequency had a shorter period from the onset of epilepsy to the beginning of treatment. West syndrome is considered a subtype of IS 23 ; therefore, the results found in this article could ratify the efficacy of everolimus in the early therapy of IS.

Regarding the use of everolimus, Svarrer et al. 15 showed excellent results since all 4 participants had a reduction greater than 50% in the frequency of seizures, and half of them were free of focal seizures for a period greater than or equal to 12 months. However, despite the positive results presented in the study, we cannot attribute them only to the use of the drug because other treatments were started simultaneously, such as vagus nerve stimulation, epilepsy surgery and ketogenic diet. In addition, during the treatment period, changes were made to the antiepileptic drugs used by the four patients. Furthermore, the seizure frequency was obtained by the families through a weekly diary, which may be associated with a measurement bias.

Although these studies can give a good insight into the relation between everolimus and TSC-related epilepsy, this is a rare condition and, consequently, unicentric studies have small samples (< 20 participants), 12 13 14 15 17 which makes the results insufficient for an adequate analysis of other factors that may contribute to the outcome of refractory epilepsy treatment with everolimus. These confounding factors are mostly the different doses of everolimus, age of the participants, type of seizures, and simultaneous or previous treatments instituted for epilepsy control between studies.

Therefore, the article by Curatolo et al. 16 was the first to analyze the use of mTOR inhibitors in an expressive pediatric group (299 participants). The results presented allow the inference that the impact of the drug use is greater in early childhood (< 6 years). In addition, the study concluded that there was a greater benefit in controlling seizures with a higher everolimus dose (9–15 ng/mL) when compared to a lower dose (3–7 ng/mL).

Even though this study found better results in younger patients (< 6 years), the clinical trial used by them for analysis, the EXIST-3, 18 excluded children under 2 years of age and with untreated IS. However, most patients with TSC have the seizures onset in the 1st year of life, and the onset of IS is observed in more than ⅔ of patients aged between 5 and 24 months, 24 25 an aspect that brings the demand for relevant studies in this group, because the earlier the onset of seizures, the worse the prognosis for behavioral and cognitive development is. 20 25 Among the studies in this literature review, only the one by Samueli et al. 14 included participants with IS during treatment with everolimus.

Adverse events that occurred significantly in more than one study were aphthous or non-aphthous stomatitis, 12 13 15 16 17 nasopharyngitis, 12 14 16 and upper airway infections. 12 13 16 17 The AEs presented in the reviewed studies are similar to those previously described in the use of everolimus to treat other conditions, in which the therapeutic effect of everolimus is already well established. 26 27 Most of the AEs described were mild or moderate, with manifestations of grades-III and -IV AEs being uncommon, which is compatible with the everolimus AEs presented by another study. 28 In addition, temporary suspension of the drug or dose reduction had a positive response in controlling the AEs, reinforcing the greater therapeutic benefits despite the risks offered. 28 However, although infrequent, death can be a possible AE of everolimus use and should be reported. Thus, the safety of everolimus lies in the fact that it is a drug with known AEs, which poses little threat when closely monitored by physicians.

In conclusion, all selected studies demonstrated beneficial results regarding the efficacy and safety of everolimus in the treatment of refractory epilepsy in children with tuberous sclerosis. 12 13 14 15 16 17 Studies show a reduction in the frequency of seizures, and despite the high frequency of AEs, most of them were of low severity and tolerable (CTCAE grades I and II). However, five of the six final articles selected were studies with small samples, and the parameters for analyzing the improvement in epilepsy control and administration of everolimus were not uniform, which limited the comparison between the studies. Although the studies are not homogeneous, everolimus proved to be efficient and safe when traditional treatments failed, with some evidence of additional benefits beyond the scope of this review, such as improved psychomotor development 15 and quality of life. 13 Thus, it is reasonable to conduct randomized double-blind controlled clinical trials with larger samples to better validate the observed effects and provide greater statistical credibility.
